# Influenza B-Triggered Secondary Hemophagocytic Lymphohistiocytosis in an Adult Male: A Diagnostic Challenge

**DOI:** 10.7759/cureus.99955

**Published:** 2025-12-23

**Authors:** Shah Toufiqur Rahman, Hana Humad

**Affiliations:** 1 Acute Medicine, Royal Liverpool University Hospital, Liverpool, GBR

**Keywords:** corticosteroid therapy, cytokine storm, hemophagocytic lymphohistiocytosis, h-score, hypercoagulability, hyperferritinemia, immunosuppression, influenza b infection, secondary hlh, viral-triggered inflammation

## Abstract

Hemophagocytic lymphohistiocytosis (HLH) is a rare, life-threatening hyperinflammatory syndrome characterized by excessive immune activation and multiorgan dysfunction. We report the case of a 39-year-old Syrian male residing in the United Kingdom who presented with high-grade fever, abdominal pain, and polyarthritis. Investigations revealed bicytopenia, hyperferritinemia, and an H-score of 213, consistent with HLH. Influenza B infection was identified as the precipitating factor after extensive evaluation excluded malignancy, tuberculosis, and autoimmune disease. The patient was treated with high-dose corticosteroids and oseltamivir, leading to rapid clinical improvement and normalization of ferritin levels. He subsequently developed a left calf deep vein thrombosis, considered a thromboembolic complication related to the hyperinflammatory and hypercoagulable state of HLH, and was managed successfully with anticoagulation. This case underscores the diagnostic complexity of adult HLH and highlights the importance of recognizing viral triggers such as influenza B in atypical inflammatory presentations.

## Introduction

Hemophagocytic lymphohistiocytosis (HLH) is an aggressive hyperinflammatory syndrome caused by dysregulated activation of cytotoxic T lymphocytes and macrophages, leading to excessive cytokine release, tissue injury, and multiorgan dysfunction [1]. Although classically described in pediatric populations with inherited forms, secondary HLH is increasingly recognized in adults and is most commonly triggered by infections, autoimmune diseases, and malignancies [2]. Adult HLH is rare, with an estimated incidence of approximately 1 per 800,000 to 1,000,000 individuals per year, and remains underdiagnosed due to its nonspecific presentation and overlap with other inflammatory conditions.

Viral infections, particularly Epstein-Barr virus and cytomegalovirus, are well-established precipitants, whereas influenza-associated HLH has been described only sporadically, primarily in isolated case reports [3]. Influenza B-triggered HLH is particularly uncommon. Adult HLH frequently mimics severe sepsis or systemic rheumatologic diseases such as adult-onset Still’s disease or Behçet’s disease, often leading to diagnostic delay. Early recognition is critical, as untreated HLH can rapidly progress to multiorgan failure. We present a diagnostically challenging case of influenza B-triggered secondary HLH in an adult male, highlighting the importance of extreme hyperferritinemia and structured diagnostic tools such as the H-score in guiding timely diagnosis.

## Case presentation

A 39-year-old male, originally from Syria and residing in the United Kingdom since 2021, presented with a five-day history of high-grade fever (maximum 40.1°C), diffuse abdominal pain, vomiting, and acute inflammatory polyarthritis. Joint involvement affected the metacarpophalangeal joints and right knee, with pain, swelling, and reduced mobility. The abdominal pain was generalized and non-radiating, without associated gastrointestinal bleeding or diarrhea. His medical history included umbilical and epigastric hernia repairs, *Helicobacter pylori*-positive duodenitis with duodenal ulceration, and bilateral varicoceles. He smoked approximately seven cigarettes daily and denied alcohol or illicit drug use.

On examination, he appeared acutely unwell and febrile. Abdominal examination revealed mild generalized tenderness without guarding or rebound. There was no pain radiation, skin rash, oral or genital ulceration, or focal neurological deficit. Mild hepatosplenomegaly, supraclavicular lymphadenopathy, and a right knee effusion consistent with inflammatory synovitis were noted.

Initial laboratory investigations demonstrated anemia, thrombocytopenia, extreme hyperferritinemia, hypertriglyceridemia, hypofibrinogenemia, elevated inflammatory markers, and mild transaminitis (Table 1).

**Table 1:** Laboratory findings demonstrating bicytopenia, hyperferritinemia, and hypertriglyceridemia at presentation, with normalization on follow-up.

Parameter	Reference range	At presentation	At follow-up
White blood cell count (×10⁹/L)	4.0–11.0	2.6	5.8
Platelet count (×10⁹/L)	150–400	69	129
Ferritin (µg/L)	30–400	15,214	91
Triglycerides (mmol/L)	<1.7	4.3	1.2
Alanine aminotransferase (U/L)	<45	75	33
C-reactive protein (mg/L)	<5	128	< 1
Hemoglobin (g/dL)	13.0–17.0	11.2	13.5
Fibrinogen (g/L)	2.0–4.0	1.5	2.9
Antinuclear antibody, dsDNA, rheumatoid factor	Negative	Negative	–

**Figure 1:**
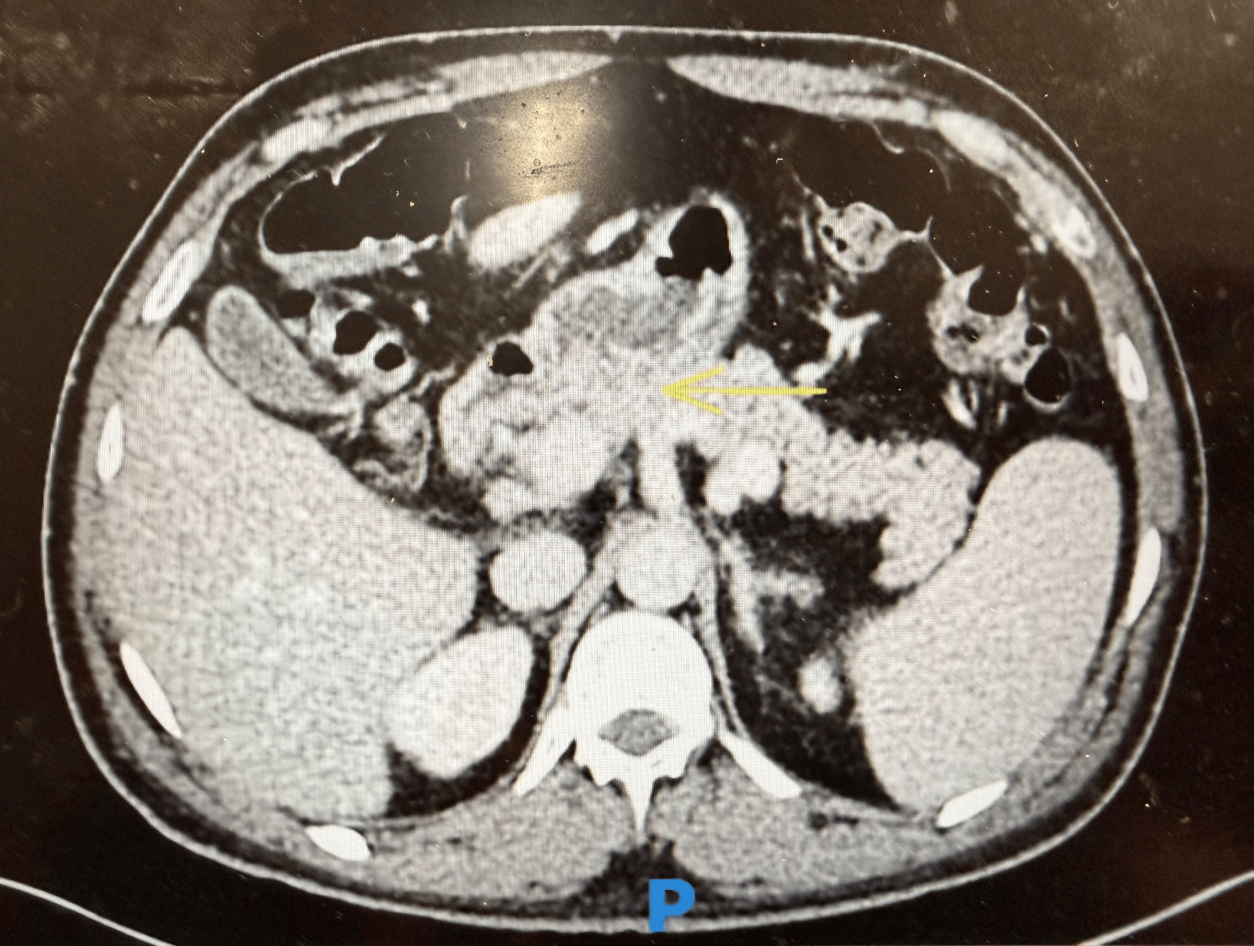
Contrast-enhanced CT of the abdomen showing duodenal fat stranding (yellow arrow).

**Figure 2:**
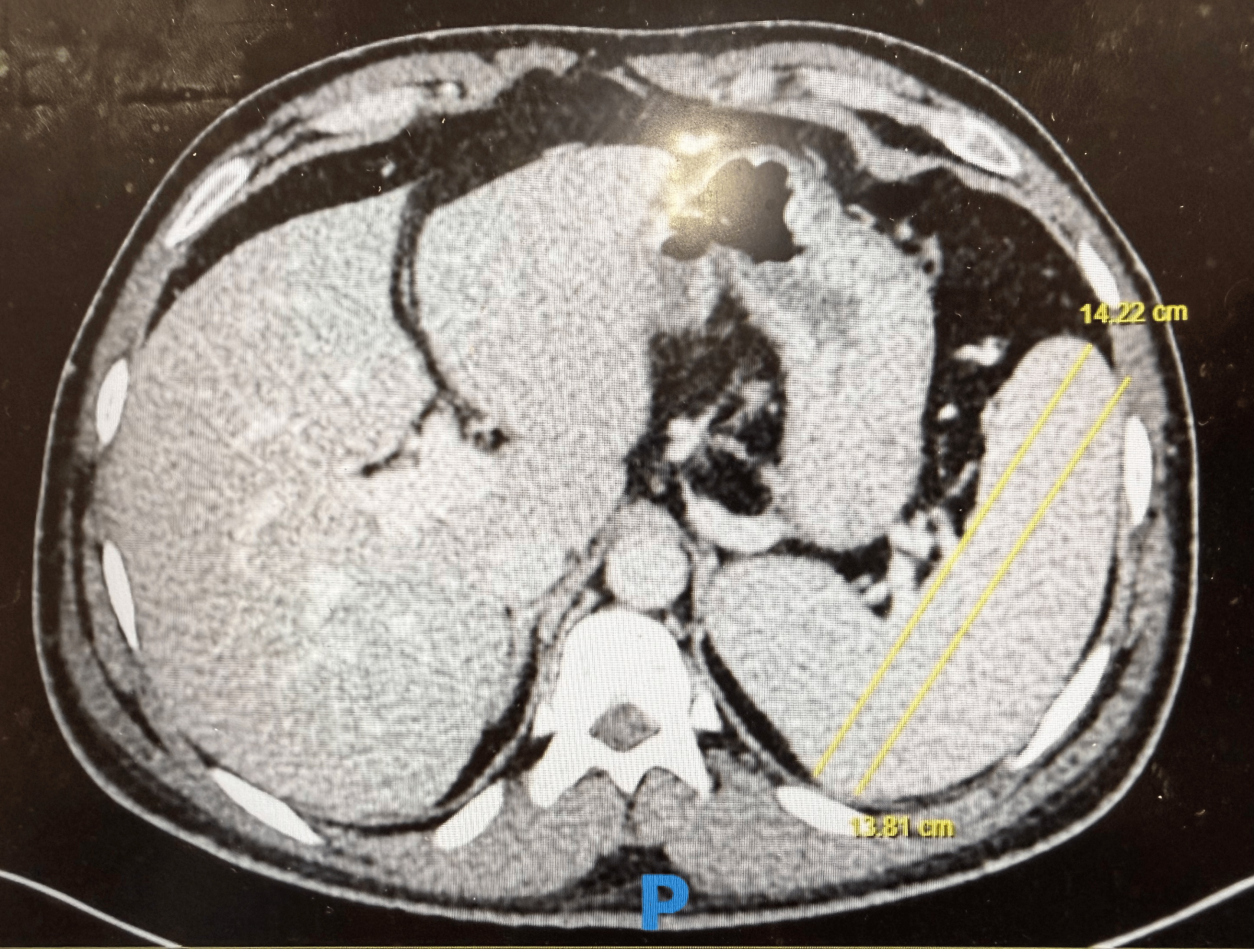
Contrast-enhanced CT of the abdomen showing splenomegaly, with splenic length measurements of 13.81 cm and 14.22 cm in two different planes.

**Figure 3:**
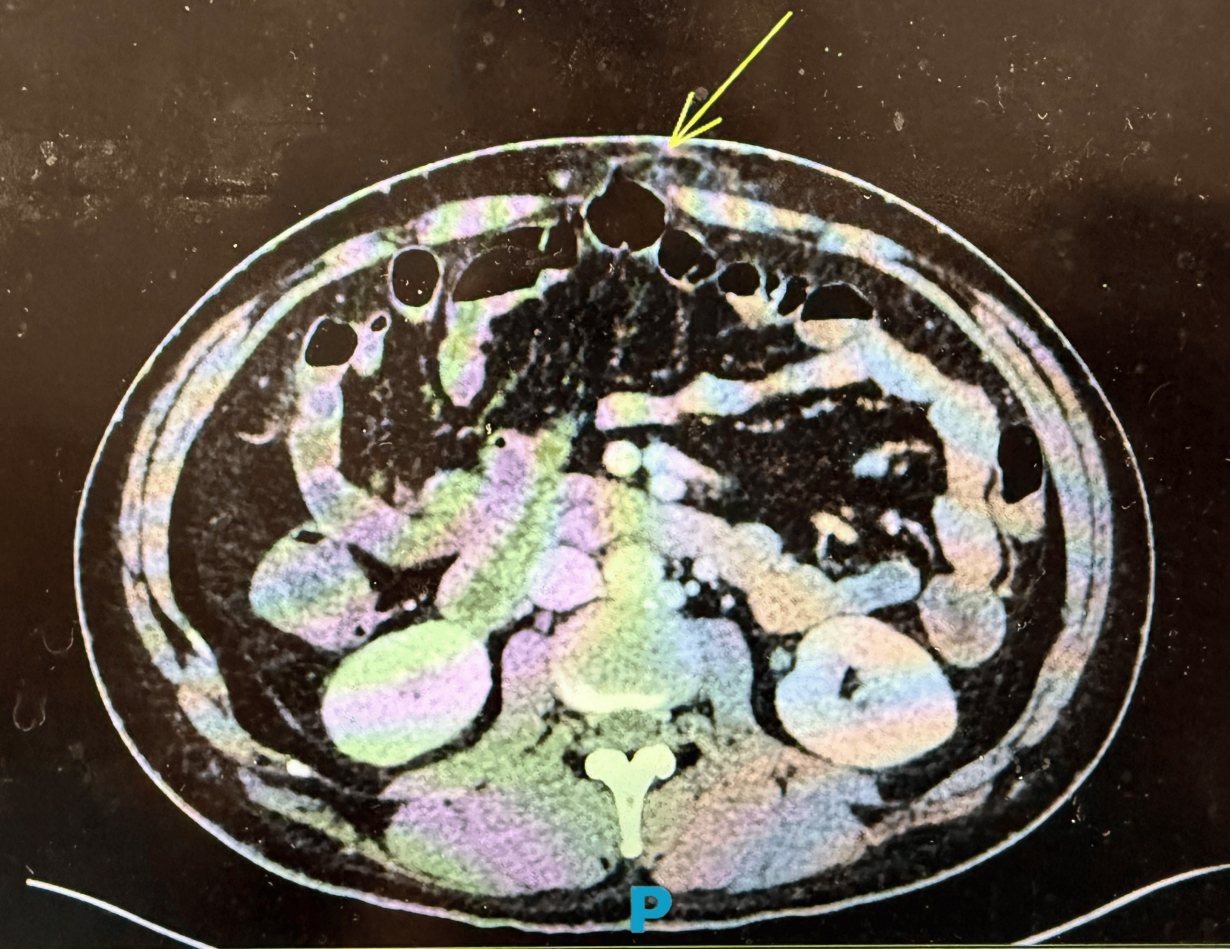
Contrast-enhanced CT of the abdomen showing an epigastric hernia without obstruction (yellow arrow).

Contrast-enhanced CT of the abdomen demonstrated duodenal fat stranding (Figure 1), splenomegaly with a maximum splenic length of 14.2 cm (Figure 2), and an epigastric hernia without evidence of obstruction (Figure 3). The presence of splenomegaly supported a systemic inflammatory or hematologic process, while imaging helped exclude focal intra-abdominal sepsis, bowel obstruction, or malignancy.

Differential diagnoses included adult-onset Still’s disease, familial Mediterranean fever, Behçet’s disease, and severe systemic infection. Extensive investigations for malignancy, tuberculosis, *Strongyloides*, *Leishmania*, *Coxiella burnetii*, and autoimmune disease were negative. Respiratory viral testing identified influenza B infection. An H-score of 213 indicated a high probability of secondary HLH.

The patient was treated with intravenous methylprednisolone (1 mg/kg/day) for three days, followed by oral prednisolone, alongside oseltamivir and gastroprotection. Empirical broad-spectrum antibiotics were discontinued when cultures remained negative. Fever resolved within 48 hours, ferritin levels declined rapidly, and cytopenias normalized.

During recovery, he developed a left-calf deep vein thrombosis, attributed to the hyperinflammatory and hypercoagulable state associated with HLH and corticosteroid exposure. He was successfully treated with therapeutic anticoagulation. At follow-up, ferritin was 91 µg/L, platelet count 129 × 10⁹/L, and C-reactive protein <1 mg/L. He remained clinically stable and was commenced on methotrexate for residual inflammatory arthritis.

## Discussion

Adult-onset HLH remains a major diagnostic challenge due to its rarity and nonspecific clinical presentation, which frequently overlaps with severe sepsis and systemic autoimmune disease [1-5]. In this case, the combination of persistent high-grade fever, inflammatory arthritis, abdominal pain, and markedly elevated inflammatory markers initially suggested infectious or rheumatologic etiologies, contributing to diagnostic uncertainty and delay.

Extreme hyperferritinemia (>10,000 µg/L) is a key diagnostic clue in HLH and should prompt urgent consideration of this diagnosis, particularly when accompanied by cytopenias, hypertriglyceridemia, and hypofibrinogenemia [6,7]. In adults, the H-score provides a validated and practical tool to estimate the probability of HLH and guide early diagnostic decision-making [4]. In this patient, an H-score of 213 supported a high likelihood of secondary HLH and helped justify prompt initiation of immunosuppressive therapy while alternative diagnoses were actively excluded.

Although Epstein-Barr virus is the most commonly reported viral trigger for HLH, influenza-associated HLH remains rare and underrecognized, particularly in adults [5,8,9]. Influenza B-triggered HLH has been described primarily in isolated case reports, which may contribute to low clinical suspicion and delayed diagnosis. The overlap of influenza-related systemic symptoms with autoimmune and inflammatory syndromes further complicates recognition, as illustrated in this case.

The pathophysiology of HLH involves dysregulated immune activation with excessive production of pro-inflammatory cytokines, including interferon-γ, interleukin-6, and tumor necrosis factor-α, resulting in macrophage activation, cytopenias, hepatic dysfunction, and coagulopathy [10]. Early immunosuppressive therapy is essential to prevent progression to multiorgan failure. Corticosteroids remain the first-line treatment for secondary HLH, while etoposide or biologic agents such as anakinra are reserved for refractory or relapsing disease [10,11]. The rapid clinical and biochemical response observed in this patient supports the effectiveness of early corticosteroid therapy in infection-associated HLH.

The development of deep vein thrombosis in this case highlights the prothrombotic risk associated with HLH. Inflammatory endothelial activation, cytokine-mediated hypercoagulability, and coagulation factor abnormalities contribute to an increased risk of both thrombotic and bleeding complications [11-13]. Clinicians should therefore maintain a high index of suspicion for thromboembolic events during both the acute illness and recovery phases.

Given the diagnostic complexity, overlap with infection and autoimmune disease, and potential for rapid clinical deterioration, a multidisciplinary approach involving hematology, infectious disease, and rheumatology specialists is critical for timely diagnosis and optimal management of adult HLH [14].

## Conclusions

HLH should be considered in adults presenting with persistent fever, cytopenias, and markedly elevated ferritin levels when common infectious and autoimmune causes have been excluded. Influenza B, although rare, may act as a trigger and mimic systemic inflammatory disorders, leading to diagnostic delay. This case underscores the importance of structured diagnostic assessment and early recognition. While corticosteroid therapy was associated with a favorable outcome in this patient, further studies are required to better define optimal management strategies and complication risks in viral-triggered HLH.
